# Elucidating the Role of *CNOT2* in Regulating Cancer Cell Growth via the Modulation of p53 and c-Myc Expression

**DOI:** 10.3390/cimb47080615

**Published:** 2025-08-04

**Authors:** Jihyun Lee, Ju-Ha Kim, Yu Jin Lee, Je Joung Oh, Yeo Jeong Han, Ji Hoon Jung

**Affiliations:** 1 CytoGen lnc., Songpa-gu, Seoul 05610, Republic of Korea; 2 Thrive Foot and Ankle Inc., Chino, CA 91710, USA; 3 College of Korean Medicine, Kyung Hee University, Seoul 02447, Republic of Korea

**Keywords:** *CNOT2*, p53, c-Myc, cancer

## Abstract

*CNOT2*, a central component of the CCR4-NOT transcription complex subunit 2, plays a pivotal role in the regulation of gene expression and metabolism. *CNOT2* is involved in various cellular processes, including transcriptional regulation, mRNA deadenylation, and the modulation of mRNA stability. *CNOT2* specifically contributes to the structural integrity and enzymatic activity of the CCR4-NOT complex with transcription factors and RNA-binding proteins. Recent studies have elucidated its involvement in cellular differentiation, immune response modulation, and the maintenance of genomic stability. Abnormal regulation of *CNOT2* has been implicated in a spectrum of pathological conditions, including oncogenesis, neurodegenerative disorders, and metabolic dysfunctions. This review comprehensively examines the interplay between *CNOT2* and p53, elucidating their collaborative and antagonistic interactions in various cellular contexts. *CNOT2* is primarily involved in transcriptional regulation, mRNA deadenylation, and the modulation of mRNA stability, thereby influencing diverse biological processes such as cell proliferation, apoptosis, and differentiation. Conversely, p53 is renowned for its role in maintaining genomic integrity, inducing cell cycle arrest, apoptosis, and senescence in response to cellular stress and DNA damage. Emerging evidence suggests that *CNOT2* can modulate p53 activity through multiple mechanisms, including the regulation of p53 mRNA stability and the modulation of p53 target gene expression. The dysregulation of *CNOT2* and p53 interactions has been implicated in the pathogenesis and progression of various cancers, highlighting their potential as therapeutic targets. Additionally, *CNOT2* regulates c-Myc, a well-known oncogene, in cancer cells. This review shows the essential roles of *CNOT2* in maintaining cancer cellular homeostasis and explores its interactions within the CCR4-NOT complex that influence transcriptional and post-transcriptional regulation. Furthermore, we investigate the potential of *CNOT2* as a biomarker and therapeutic target across various disease states, highlighting its significance in disease progression and treatment responsiveness.

## 1. Introduction

Cancer is a multifaceted disease arising from genetic and epigenetic alterations that drive uncontrolled cell proliferation, invasion, and metastasis. Benign tumors are typically characterized by slow growth and a lack of invasive properties, preventing them from infiltrating nearby tissues or spreading to distant organs. However, depending on their size, anatomical location, and the risk of malignant transformation, medical intervention, such as surgical removal, may be warranted. Certain benign tumors, including adenomas and particular meningiomas, have the potential to cause clinical complications by exerting pressure on adjacent structures or progressing into malignant forms. In contrast, malignant tumors exhibit rapid proliferation, local tissue invasion, and the capacity for distant metastasis through the bloodstream or lymphatic system. These aggressive characteristics necessitate prompt therapeutic interventions, including surgery, chemotherapy, and radiation therapy, to control disease progression and improve patient outcomes [1,2]. Recent studies suggest that cancer progression is influenced not only by genetic mutations but also by regulatory interactions between key oncogenic and tumor suppressor pathways. Among these, *CNOT2* (CCR4-NOT Transcription Complex Subunit 2) and p53 play *CNOT2* (CCR4-NOT Transcription Complex Subunit 2) and p53 are pivotal regulators in cancer biology, influencing tumor progression, metastasis, and treatment response.

*CNOT2* is a core component of the CCR4-NOT complex, a multi-subunit protein complex involved in mRNA degradation, transcriptional regulation, and protein ubiquitination. While the CCR4-NOT complex has been extensively studied, recent research has highlighted its emerging role in cancer pathophysiology. *CNOT2* has been identified as a critical regulator of oncogenic pathways, contributing to tumor progression, immune evasion, and resistance to anticancer therapies [3,4]. Notably, studies indicate that *CNOT2* modulates c-Myc stability, thereby impacting metabolic reprogramming and tumor cell proliferation [3,5].

The tumor suppressor p53 is one of the most extensively investigated genes in cancer research. Across diverse scientific disciplines—including molecular biology, pharmacology, biochemistry, and genetics—ongoing studies continue to elucidate novel regulatory mechanisms of p53 [1,2,6]. p53 functions as a key tumor suppressor by responding to cellular stressors such as DNA damage, ribosomal stress, and oncogenic activation. It orchestrates diverse cellular processes, including apoptosis, cell cycle arrest, and senescence, while also inhibiting angiogenesis, tumor cell migration, and metastasis [7,8,9,10,11,12].

However, TP53 mutations are among the most common genetic alterations in human malignancies, contributing to loss of tumor suppressive function and gain-of-function oncogenic activity [13,14]. Recent findings indicate that certain p53 mutations not only abolish tumor-suppressive activities but may also confer oncogenic properties that promote cancer cell survival and metastasis [14].

Recent research has uncovered a direct regulatory relationship between *CNOT2* and p53, providing critical insights into tumorigenesis. *CNOT2* depletion has been shown to induce apoptosis in p53 wild-type (WT-p53) cancer cells, whereas it exhibits an opposing effect in p53-null cells, suggesting a context-dependent role in tumor progression [14,15,16]. While *CNOT2* has been implicated in transcriptional repression and RNA metabolism, its role in EMT and metastasis is only beginning to be elucidated. Recent studies suggest potential involvement in regulating cell motility and EMT-related gene networks [17,18]. Accumulating evidence also suggests that the interaction between *CNOT2* and p53 may modulate chemotherapy sensitivity, with studies demonstrating that *CNOT2* inhibition enhances the efficacy of chemotherapeutic agents such as Doxorubicin and 5-Fluorouracil (5-FU) [14].

Furthermore, recent investigations suggest that *CNOT2* contributes to immune evasion in tumors by regulating cytokine production and T-cell infiltration within the tumor microenvironment. This growing body of evidence underscores the potential of targeting *CNOT2*, either independently or in combination with p53-restoring therapies, as a novel therapeutic strategy for cancer treatment.

c-Myc is a master regulator of transcription, cell cycle progression, and metabolism, and is one of the most frequently deregulated oncogenes in human cancers. It promotes the expression of genes involved in ribosomal biogenesis, glycolysis, and survival [13]. c-Myc also enhances ribosomal biogenesis and protein synthesis, whereas c-Myc is regulated by ribosomal proteins as feedback regulators [19].

Recent studies highlight the importance of transcriptional and post-transcriptional regulators in shaping the tumor microenvironment and determining cancer cell fate. *CNOT2*, as a core component of the CCR4–NOT complex, has emerged as a key modulator of gene expression programs that influence proliferation, apoptosis, and metabolic adaptation in tumor cells. Rather than focusing on general tumor classifications, this review emphasizes the functional role of *CNOT2* in cancer progression and its interplay with major regulatory molecules such as p53 and c-Myc.

## 2. Oncogenic Functions of *CNOT2* in Cancer Cells

**Figure 1:**
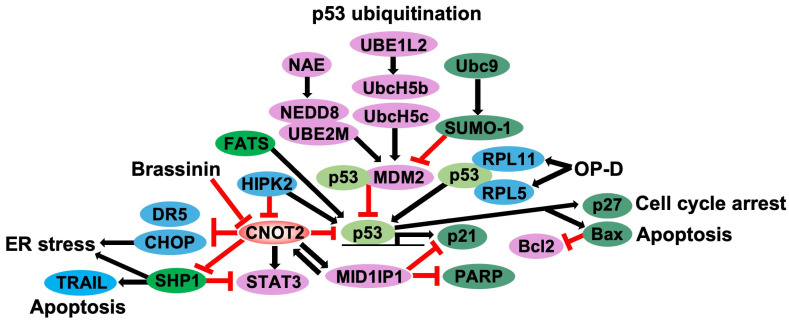
The scheme of *CNOT2* and p53-related gene regulation. *CNOT2* increases MID1IP1 and STAT3, whereas *CNOT2* blocks cell cycle arrest, apoptosis, and ER stress pathways, regulating DR5, CHOP, SHP1, and p53 genes (Black arrows). MID1IP1 upregulates *CNOT2* expression and suppresses p21 and PARP signaling (Black arrows). Brassinin, a single compound, inhibits *CNOT2*. Ophiophogonin D (OP-D) regulates RPL5 and RPL11 to increase p53 expression (Red lines). HIPK2 activates p53 and reduces *CNOT2* expression. p53 ubiquitination regulates MDM2 activation with neddylation (NEDD8) and Sumoylation (SUMO-1; Red lines).

Cancer can occur when a proto-oncogene is mutated and turned into an oncogene, and cells divide and multiply out of control. Here, the oncogene is a mutant gene with the potential for tumor development. Before an oncogene is mutated, it is called a proto-oncogene, and it plays a role in dividing normal cells [20]. *CNOT2* has been implicated in oncogenic processes, including mRNA degradation, apoptosis regulation, and ER stress response, highlighting its role in cancer progression. When *CNOT2* is depleted, the formation of P-body presumed to be mRNA degradation is suppressed. In addition, CHOP mRNA transcription is upregulated to induce endoplasmic reticulum (ER) stress and caspase-dependent apoptosis [21]. In hepatocellular carcinoma cell lines (HepG2 and Huh7), *CNOT2* deficiency was found to enhance the antitumor effects of MID1IP1 depletion while reducing apoptosis markers, suggesting a functional interplay between *CNOT2* and MID1IP1 in liver cancer cells [17]. Further studies were conducted based on previous papers that confirmed a close relationship between *CNOT2* and *MID1IP1*. It was confirmed that the inhibition of *CNOT2* activates *p53* and induces apoptosis through *MID1IP1* [12]. In non-small cell lung cancer (NSCLC) cells (H1299), atorvastatin treatment was found to be associated with apoptotic and autophagic pathways, where *CNOT2* played a crucial regulatory role. The overexpression of HA-*CNOT2* suppressed apoptotic and autophagic processes, whereas *CNOT2* depletion enhanced these functions [16]. Moreover, in NSCLC cell lines (A549, H1299, H596, and H460), *CNOT2* depletion was linked to apoptosis, SHP1 activation, and ER stress induction. In TRAIL-resistant lung cancer cells, *CNOT2* knockdown sensitized cells to TRAIL-mediated apoptosis by modulating SHP1-STAT3 signaling and activating death receptor 5 (DR5) through intracellular stress responses [22]. Recent studies have also investigated the role of *CNOT2* in drug response. Ophiopogon (OP) is a substance extracted from the root mass of ophiopogon japonica and has an anticancer effect. Ophiophogonin D (OP-D) of Ophiophogon (OP) induced *p53* expression through ribosomal proteins L5 and L11 and inhibited c-Myc expression independently of *CNOT2* capacity. It is notable here that the depletion of *CNOT2* increased the Opiopogon D (Op-D) effect on *c-Myc* in colon cancer cells [23]. Similarly, Brasinin extracted from Chinese cabbage was dose- and time-dependently correlated with *CNOT2* and *p53* expression in HCT116 colorectal cancer cells, further underscoring the therapeutic potential of targeting *CNOT2* in cancer treatment [18]. In summary, *CNOT2* is implicated in cancer progression through its roles in mRNA degradation, apoptosis regulation, and ER stress response. The depletion of *CNOT2* enhances apoptosis via interactions with MID1IP1, p53 activation, and modulation of SHP1-STAT3 signaling pathways in various cancers (Figure 1).

## 3. *CNOT2* Promotes Cancer Cell Proliferation, Angiogenesis, and EMT

**Figure 2:**
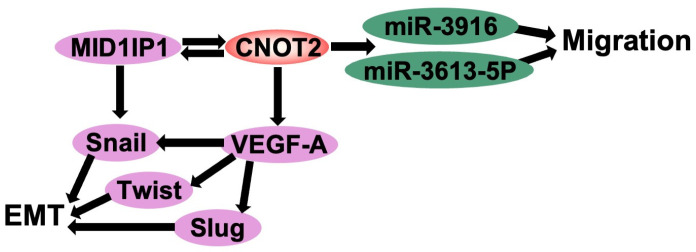
The scheme of *CNOT2* and EMT-related gene regulation. *CNOT2* increases MID1IP1, VEGF-A, Snail, Twist, Slug, has-miR-3916, and has-miR-3613-5p to elevate the migration and EMT of cancer cells (Black arrows).

A tumor is defined as an abnormal mass of cells that evades normal cell cycle regulation and undergoes uncontrolled proliferation. Tumors are broadly classified into malignant and benign types [7]. Benign tumors are generally well-differentiated, slow-growing, and non-invasive, lacking the ability to metastasize to distant organs. However, depending on their size, location, and potential for malignant transformation, medical intervention may be required. In contrast, malignant tumors proliferate uncontrollably, invade surrounding tissues, and metastasize through the bloodstream or lymphatic system, necessitating prompt therapeutic intervention. [9]. In breast cancer, angiogenesis and epithelial–mesenchymal transition (EMT) are critical processes that drive tumor progression and metastasis. Vascular endothelial growth factor A (VEGF-A) is a key regulator of angiogenesis, stimulating new blood vessel formation to sustain tumor growth and enhance its aggressiveness [8]. Recent studies have demonstrated that *CNOT2*, a core subunit of the CCR4-NOT complex, is a crucial modulator of these oncogenic processes [16,17,18]. *CNOT2* was found to be overexpressed in breast cancer cell lines (MCF-7 and MDA-MB-231), promoting tumor proliferation and metastasis. Conversely, *CNOT2* depletion led to reduced motility in MDA-MB-231 cells, the downregulation of proliferation-related gene expression, and the suppression of VEGF signaling [15]. The reduction in VEGF expression upon *CNOT2* depletion led to decreased angiogenic potential, suggesting that *CNOT2* is a critical upstream regulator of VEGF-A-mediated angiogenesis. EMT is characterized by the loss of epithelial markers and the acquisition of mesenchymal traits, enabling cancer cells to invade and metastasize. EMT-related genes, including Snail, Slug, and Twist, were found to be significantly downregulated in MDA-MB-231 cells lacking *CNOT2*. Furthermore, miRNA profiling of these cells revealed that has-miR-3613-5p and has-miR-3916 play critical roles in relieving migration inhibition. These miRNAs may serve as downstream effectors of *CNOT2*, acting as regulatory nodes in breast cancer cell migration and EMT [24]. Given its regulatory involvement in tumor proliferation, angiogenesis, and metastasis, *CNOT2* emerges as a potential therapeutic target for breast cancer. Inhibiting *CNOT2* could effectively suppress tumor growth and metastatic progression, providing a multifaceted approach to cancer treatment. Future studies should focus on elucidating the precise molecular mechanisms by which *CNOT2* modulates oncogenic pathways and developing targeted inhibitors to disrupt its function (Figure 2).

## 4. Knockdown of *CNOT2* Induces p53 Activation

Numerous reports in relation to p53 have been available for 40 years since its first discovery, the year 1979 [25]. p53 plays a critical role as a guardian of cell genome, responding to DNA double strand breaks and regulating various target genes as a transcription factor [26]. It has been determined that p53 mediates tumor-suppressing mechanisms such as apoptosis and cell cycle [27]. The half-life of the p53 protein is tightly regulated and significantly prolonged in response to cellular stress signals such as DNA damage [28]. One of the most well-known mechanisms regulating p53 is autoregulatory feedback loops with MDM2 [29]. The N-terminal domain of MDM2 binds to N and C terminal sites of p53 and regulates its proteolysis [30]. In this section, recent studies about p53 were organized including MDM2 as well as ribosomal proteins, ubiquitylation, and neddylation and un-masked in association with interactive characteristics between p53 and *CNOT2*.

### 4.1. Regulation of p53-MDM2 Loop by Ribosomal Proteins

A total of 47S/45S pre-rRNA, RP-encoding mRNAs, 5S rRNA, non-ribosomal factors, and small nucleolar RNAs were recruited, comprising the 90S pre-ribosomes in the nucleolus, and then matured to 60S and 40S subunits for protein translation [31]. Recently, various ribosomal proteins have been studied to determine what inhibits and activates p53. It has been found that ribosomal biogenesis stimulates p53 via the suppression of MDM2 E3 ligase in combination with ribosomal proteins including RPL5, RPL11, RPL23 [32], S7 [33], S14 [34], RPS25 [35], RPS15 [36], RPL22 [37], RPS2 [38], and RPL34 [39]. On the other hand, p53 was shown to be activated upon disruption of specific ribosome proteins containing S27L [40], RPL37 [41], RPL31 [42], RPL4 [43], and RPL40 [44] (Table 1). This regulatory relationship is particularly relevant in the context of *CNOT2*, as recent data indicate that *CNOT2* knockdown leads to the upregulation of RPL5 and RPL11. These proteins stabilize p53 by blocking its degradation via MDM2, providing a mechanistic link between *CNOT2* depletion and enhanced p53-mediated tumor suppression.

### 4.2. Regulation of p53-MDM2 Loop: Ubiquitylation and Neddylation

Fuchs et al. found that p53 is degraded by MDM2 whose degradation was downregulated when sites between 150 and 461 aa were deleted [45]. Next, specific sites of MDM2 regulating p53 were determined to be the main residues between 202 and 302 functioning as MDM2 RING (really interesting genes) finger [46]. Also, it was identified that p53 binds this region of MDM2, which acts as an E3 ubiquitin ligase and is then subsequently degraded by the 26S proteasome [47]. The ubiquitin-conjugating enzymes (E2s) as well as the E3 ligase MDM2 also support MDM2 auto-ubiquitination of p53. Mark et al. showed that depletion of the ubiquitin-conjugating enzyme UbcH5B and -C inhibits ubiquitination of p53 [48]. The ubiquitin-activating enzyme (E1) UBE1L2 activates ubiquitin, transfers it onto UbcH5b, and interacts with MDM2 and ubiquitinate p53 [49]. In addition, many E2 that regulate the ubiquitination of p53 have been consistently discovered and demonstrated. In addition, various mechanisms were also identified. FATS promoted the stabilization of p53 as an E2-independent ubiquitin ligase [50]. Binding SUMO1 to Mdm2 was followed by the conjugation of Su-mo-1 to MDM2 by Ubc9 [51]. It was determined that the neddylation mechanism similar to ubiquitination mediates the ubiquitin–proteasome system as an important signaling pathway. Neddylation is caused by neural precursor cell-expressed, developmentally downregulated protein 8 (NEDD8) E1 activating enzyme (NAE), and NEDD8 E2/E3 enzymes with NEDD8. Interestingly, in our previous study, it was documented that NEDD8 E2 enzyme UBE2M blocks p53 while binding to MDM2 and RPL11 [52]. These classical mechanisms of p53 regulation through MDM2 and ribosomal proteins provide a crucial framework for understanding the emerging role of *CNOT2*. Notably, *CNOT2* knockdown has been shown to enhance the expression of ribosomal proteins such as RPL5 and RPL11, both of which are known to inhibit MDM2 and stabilize p53. This suggests that *CNOT2* may indirectly suppress p53 activity by modulating the availability or function of these ribosomal protein regulators.

### 4.3. CNOT2 Knockdown Induces p53 in Cancer Cells

Building on this regulatory framework, recent evidence indicates that *CNOT2* directly influences the stability and activity of p53 in a ribosomal protein-dependent manner. In colorectal cancer cells, *CNOT2* knockdown leads to the activation of p53 and apoptosis, supporting its role as an upstream modulator of the p53 axis through both transcriptional and translational mechanisms. *CNOT2* has been identified as a key regulator in various cancer-related pathways. Previous studies have demonstrated that *CNOT2* plays a critical role in atorvastatin-induced apoptosis in non-small cell lung cancer cells [16]. Additionally, *CNOT2* has been reported to promote breast cancer cell proliferation and angiogenesis [15]. Moreover, it has been implicated in liver cancer progression through its regulation of c-Myc via ribosomal proteins [17].

Given these findings, we postulate that *CNOT2* knockdown may contribute to p53 activation, thereby promoting apoptosis in cancer cells. Consistent with this hypothesis, Rosa Puca et al. reported that HIPK2, a well-established activator of p53, limits *CNOT2*-dependent mRNA decay, leading to increased p53 stability and activity [53]. Further supporting this, bioinformatics analysis has demonstrated a significant correlation between *CNOT2* expression and the p53 signaling pathway, suggesting a regulatory association between these two factors [38]. Several studies have employed RNA interference (siRNA) to investigate the role of *CNOT2* in p53 regulation. In colorectal cancer cells, siRNA-mediated knockdown of *CNOT2* resulted in prolonged p53 half-life and enhanced apoptosis. Additionally, co-immunoprecipitation (co-IP) experiments have been used to detect protein–protein interactions between *CNOT2* and p53 regulatory elements, including MID1IP1 and ribosomal proteins such as RPL5 and RPL11. Moreover, transcriptomic and pathway enrichment analyses have demonstrated a strong positive correlation between *CNOT2* expression and the downregulation of p53 signaling pathways across multiple cancer datasets. These findings underscore the relevance of both direct and indirect regulatory interactions between *CNOT2* and p53 in cancer pathophysiology.

Moreover, experimental evidence has shown that silencing *CNOT2* enhances TRAIL sensitivity in cancer cells, thereby counteracting mechanisms that allow tumor cells to evade p53-mediated apoptosis [37]. Recent studies have also indicated that the depletion of *CNOT2* results in p53 accumulation and heightened apoptotic activity in colorectal cancer cells, as evidenced by a prolonged p53 half-life in *CNOT2*-deficient cells compared to controls [39]. Additionally, MID1IP1 has been identified as a crucial modulator of p53 stability, acting through its interaction with *CNOT2*.

Collectively, these findings suggest that *CNOT2* plays an essential role in regulating p53-mediated apoptosis in cancer cells. The suppression of *CNOT2* not only stabilizes p53 but also enhances its pro-apoptotic function, highlighting its potential as a therapeutic target for cancer treatment.

### 4.4. Modulation of p53 Activity Through CNOT2 Inhibition as Potential Anticancer Strategy

Accumulating evidence suggests a functional interplay between *CNOT2* and the tumor suppressor p53 in cancer cells. Experimental studies have demonstrated that the downregulation of *CNOT2* can influence p53-associated signaling pathways. In particular, treatment with Brassinin, a phytoalexin compound, was reported to reduce the expression levels of both *CNOT2* and p53, and its co-treatment with Doxorubicin led to enhanced cytotoxic effects in vitro, implying a potential for combination-based therapeutic strategies [18].

A variety of chemotherapeutic agents are known to exert their effects through the activation of the p53 pathway. Among them, Doxorubicin (Adriamycin) [54], 5-Fluorouracil [55,56], Docetaxel (Taxotere), and Cisplatin [57,58] have been approved for clinical use and are widely applied in standard cancer treatment protocols. Although specific small-molecule inhibitors of *CNOT2* have not yet been developed, experimental approaches such as siRNA-mediated gene silencing have been utilized to investigate its biological function and therapeutic relevance (Figure 1).

Further studies are warranted to elucidate the molecular mechanisms through which *CNOT2* modulates p53 stability and activity, and to determine whether targeting *CNOT2* could enhance the responsiveness to conventional p53-based therapies. Additionally, the identification of biomarkers associated with *CNOT2* expression or activity may contribute to the development of more personalized and effective therapeutic interventions.

## 5. *CNOT2* Regulates c-Myc Expression via Ribosomal Proteins

### 5.1. Function of c-Myc in Cancer Cells

**Figure 3:**
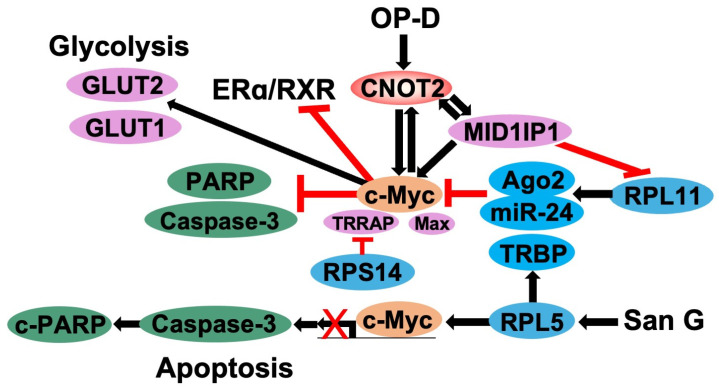
The scheme of *CNOT2* and c-Myc-related gene regulation. *CNOT2* increases c-Myc, MID1IP1, GLUT1, and GLUT2 expression, inhibiting apoptosis and stimulating glycolysis (Black arrows), whereas *CNOT2* blocks ligands of estrogen receptor alpha (ERα) and retinoid X receptor (RXR; Red lines). MID1IP1 and c-Myc upregulate *CNOT2* expression. RPL11, RPL5, and RPS14 block c-Myc through Ago2/miR-24, TRBP, and RPS14 signaling. Sanggenon G (San G) induces RPL5 signaling (Red lines).

c-Myc is a transcription factor belonging to the basic helix–loop–helix (bHLH) family and is mainly present in the nucleus of the cell. In addition, c-Myc is overexpressed in cancer cells and regulates cell growth, differentiation, metabolism, and apoptosis [59]. The regulation of c-Myc expression is primarily dependent on its protein stability. c-Myc is a short-lived protein, with a half-life of approximately 30 min for protein, making it an efficient mechanism for gene regulation [60]. When genetic alterations in c-Myc occur in non-coding regulatory regions rather than protein coding sequences, the regulation of c-Myc expression causes more cell proliferation effects [61]. c-Myc plays a key role in promoting the transcription of genes encoding essential proteins. Since these genes are involved in ribosomal biogenesis, ribosomal protein translation, and overall ribosomal function, c-Myc can activate ribosomal biogenesis and protein synthesis. This is the primary mechanism through which c-Myc regulates cell growth and proliferation [62]. c-Myc is considered a promising target for cancer treatment because it is highly active in various types of cancer (Figure 3). Consequently, drugs targeting c-Myc can be developed as potential therapies for a wide range of malignancies [63].

### 5.2. Regulation of c-Myc by Ribosomal Proteins

Certain ribosomal proteins can be regulated by c-Myc, which is responsible for promoting ribosomal RNA and protein transcription to facilitate ribosomes biogenesis [62]. Since ribosomal proteins are affected by oncogenic factors and dysregulated translation processes, emerging evidence suggests that mutations in ribosomal proteins are critically involved in ribosomopathies and carcinogenesis [17]. Several ribosomal proteins affected in cancer are associated with widely known oncogenes, tumor suppressors [63]. Specifically, ribosomal proteins RPL5, RPL11, and RPS14 regulate c-Myc by recruiting it to RISC for c-Myc expression and c-Myc mRNA degradation [62].

Recently, it has been shown that one of the functions of ribosomal protein L11 (RPL11) is to act as a feedback regulator of c-Myc [64]. RPL11, a ribosomal protein, plays a key role not only in ribosome function but also in transmitting ribosomal stress signals to the p53-dependent cell cycle checkpoint by inhibiting MDM2, a major negative regulator of p53 [65]. RPL11 interacts with c-Myc in the promoter regions of c-Myc’s target genes and represses its transcriptional activity in response to ribosomal stress [62,66,67]. Additionally, RPL11 binds to miR-24 as well as c-Myc mRNA at 3′ untranslated region(3′UTR), a key component of the microRNA-induced silencing complex (miRISC) argonaute 2 (Ago2), which induces reduction by recruiting miR-24-loaded miRISCs and then degrading c-Myc mRNA [66]. The ablation of ago2 abrogated RPL11-mediated reduction in c-Myc mRNA. On the other hand, the knockdown of RPL11 significantly increases the mRNA level and stability of miR-24-mediated c-Myc mRNA in cells [65,66].

Ribosomal protein L5 (RPL5) also has a role in regulating c-Myc. A study on the effects of SanG found that it inhibits the proliferation of non-small cell lung cancer cells and induces cell apoptosis through a combination of caspase3 activation and RPL5-mediated inhibition of c-Myc [68]. RPL5 mediates the targeting of c-Myc mRNA via miRNAs by binding to the 3′UTR of c-Myc mRNA, the HIV-1 TAR RNA Binding Protein (TRBP), and Ago2. The knockdown of RPL5 induced c-Myc expression at both the mRNA and protein levels, while the overexpression of RPL5 suppressed c-Myc expression and activity [62].

The ribosomal proteins RPL5 and RPL11 function together to co-suppress c-Myc expression. RPL5 cooperates with RPL11 to recruit the RNA-induced silencing complex (RISC) to c-Myc mRNA, facilitating its degradation and thereby suppressing c-Myc activity [62]. Concerning the depletion of MID1IP1 in HepG2, Huh7 cells activated RPL5 and RPL11 and reduced c-Myc. The upregulation of RPL5 or RPL11 is regulated through the depletion of MID1IP1, and the depletion of RPL5 and RPL11 activates c-Myc in MID1IP1-depleted HepG2 and Huh7 cells, suggesting that RPL5 and RPL11 are responsible for the regulation of c-Myc as tumor suppressors [17].

Also, RPS14 is a ribosomal protein that regulates c-Myc. RPS14 prevents c-Myc and its cofactor TRRAP from binding to target gene promoters. A deficiency of RPS14 regulates both the mRNA and protein levels of c-Myc. The Myc homology box II (MBII) of the oncogene c-Myc and C-terminal basic helix–loop–helix leucine zipper (bHLH-LZ) domains interact with RPS14. When c-Myc transcription is inhibited by RPS14, c-Myc-driven cell proliferation is also inhibited. RPS14, which directly inhibits transcriptional activity and mediates mRNA degradation via miRNAs, abrogates the function of c-Myc [69]. Collectively, ribosomal proteins such as RPL5, RPL11, and RPS14 regulate c-Myc by influencing its transcriptional activity and promoting degradation through interactions with the miRNA-induced silencing complex (RISC). RPL5 and RPL11 collaborate to suppress c-Myc expression via miRNA-mediated mRNA degradation, thus acting as tumor suppressors. RPS14 inhibits c-Myc activity by blocking its binding to gene promoters and facilitating its degradation, thereby restraining c-Myc-driven cancer cell proliferation.

### 5.3. Knockdown of CNOT2 Effects c-Myc Expression

In the metabolic processes of eukaryotic cells, the CCR4-NOT complex controls several steps in mRNA production [10]. *CNOT2*, one of the nine subunits of the CCR4-NOT complex, acts to regulate transcription and translation, but can also work as on oncogene to promote proliferation, lipid metabolism, angiogenesis, and autophagy [11,13,19].

Recent studies have shown that *CNOT2* functions as an oncogene when proliferation and tumor angiogenesis was inhibited through Vascular Endothelial Growth Factor (VEGF) signaling are suppressed in *CNOT2*-suppressed human cancer cells (Figure 2) [25]. In addition, previous studies have shown that *CNOT2* can regulate the expression of c-Myc and thus induce apoptosis in cancer cells. In tumor tissue, *CNOT2* is overexpressed, and in pancreatic cancer, *CNOT2* regulates the expression of c-Myc. And there have also been studies suggesting that liver cancer growth can regulate c-Myc in the liver, mediated by *CNOT2* [17]. Furthermore, researchers studied the oncogenic potential of MID1IP1 in hepatocellular carcinoma cell (HCC) growth in relation to ribosomal protein L5 and L11 and *CNOT2*-mediated c-Myc signaling and found that *CNOT2* knockdown could further inhibit the downregulation of MID1IP1 by c-Myc. The inhibition of *CNOT2* potentiated the antitumor effect produced by reduced MID1IP1 and downregulated c-Myc, pro-caspase3, and pro-PARP in MID1IP1-depleted HepG2, Huh7, and HCT116 cells. MID1IP1 supports the cooperation of proto-oncogene c-Myc, mediated by RPL5, RPL11, and *CNOT2*, which acts as a potent oncogene molecule, in the progression of liver cancer [17]. Moreover, *CNOT2* can also inhibit the ligand-dependent transcriptional activation of ERα and retinoid X receptor (RXR) target genes such as c-Myc in MCF-7 human breast cancer cells [15] (Table 2).

**Figure 4:**
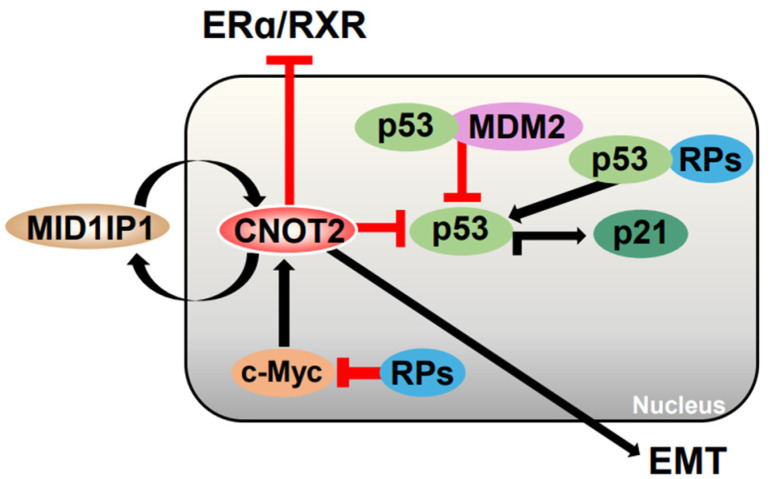
Schematic of *CNOT2* regulatory network. *CNOT2* enhances expression of MID1IP1 and promotes EMT-related pathways (Black arrows). MID1IP1 and c-Myc, in turn, upregulate *CNOT2*, forming positive feedback loop (Black arrows). Although previously omitted, *CNOT2* also regulates c-Myc expression by modulating its mRNA stability and protein turnover through ribosomal protein RPL5 and RPL11, indicated by reciprocal arrows (Red lines).

These results suggest that the knockdown of *CNOT2* may have a significant effect on the expression of c-Myc and that the colocalization of *CNOT2* and c-Myc may regulate cell death in tumors (Figure 4).

## 6. Conclusions

Recent studies have revealed the critical role of *CNOT2* in cancer progression, highlighting its involvement in multiple oncogenic processes. *CNOT2*, as a key component of the CCR4–NOT complex, influences tumorigenesis primarily through its regulation of mRNA stability and transcription. Its modulation of p53 and c-Myc activity has been shown to support cancer cell survival, proliferation, and therapy resistance.

Beyond its role in transcriptional and post-transcriptional regulation, *CNOT2* has been implicated in drug resistance, immune evasion, and metabolic reprogramming, underscoring its potential as a key factor in tumor adaptation and therapy resistance. Notably, recent studies indicate that *CNOT2* depletion enhances chemosensitivity in certain cancer types, suggesting that targeting *CNOT2* may improve treatment efficacy when combined with conventional anticancer agents.

Interestingly, recent studies demonstrate that *CNOT2* may exert opposite biological effects depending on tumor context. In breast cancer models, *CNOT2* has been shown to promote proliferation and angiogenesis through VEGF signaling [15], whereas in colorectal cancer cells, *CNOT2* depletion activates p53 signaling via MID1IP1, leading to apoptosis [12]. These findings suggest that *CNOT2* may function either as an oncogene or a tumor suppressor, contingent on cancer type and cellular environment. This tumor-type specificity highlights the importance of context-dependent investigations when evaluating *CNOT2* as a therapeutic target.

Furthermore, *CNOT2* undergoes MK2-dependent phosphorylation in response to cellular stress, which suppresses deadenylase activity and induces apoptosis [70]. These findings reveal additional layers of post-translational regulation and suggest that *CNOT2* contributes to stress responses in cancer cells.

Structurally, the CCR4–NOT complex functions as a multi-subunit regulatory platform composed of enzymatic and scaffolding components. CNOT1 acts as the central hub, organizing other subunits including *CNOT2*, CNOT3, CNOT6/6L, and CNOT7 to coordinate deadenylation, transcriptional repression, and RNA surveillance [71]. Moreover, the complex has been proposed to operate as a versatile “chaperone platform” that integrates RNA metabolism with broader cellular functions such as DNA repair, protein degradation, and chromatin remodeling [72].

Despite these advancements, several questions remain unanswered. The precise molecular mechanisms governing *CNOT2*-mediated oncogenic signaling, its potential interaction with other cancer-associated pathways, and its context-dependent roles across different tumor types require further investigation. Additionally, given the dual role of *CNOT2* in both oncogenic and tumor-suppressive mechanisms depending on the cellular context, future research should explore its function in a broader spectrum of malignancies.

Although *CNOT2* has shown promise in regulating cancer-related signaling pathways such as p53 and c-Myc, its clinical utility as a biomarker or therapeutic target remains an emerging hypothesis. Future studies incorporating transcriptomics, proteomics, and metabolomics will be essential to validate *CNOT2*’s diagnostic and prognostic potential and to uncover its broader network-level interactions in various tumor contexts.

Taken together, *CNOT2* emerges as a key regulator that integrates oncogenic signaling with transcriptional, post-transcriptional, and stress-response pathways. Given its dual role in supporting oncogenesis or enabling tumor suppression depending on the cellular context, *CNOT2* represents a compelling but nuanced target whose therapeutic exploitation will require precise, tumor-specific strategies.

## Figures and Tables

**Table 1:** Ribosomal proteins (RPs) that modulate p53 activity. Some RPs such as RPL5, RPL11, and RPS14 stabilize p53 by inhibiting MDM2, while others downregulate p53 (p53↓). These RPs are known to be upregulated following *CNOT2* knockdown, suggesting an indirect pathway through which *CNOT2* regulates p53 activity (p53↑).

RPs (p53↓)	RPs (p53↑)
S27L	RPL5
RPL37	RPL11
L31	RPL23
RPL4	S7
RPL40	S14
	RPS25
	RPS15
	RPL22
	RPS25
	RPL34

**Table 2:** Downregulated and upregulated genes by *CNOT2* and genes downregulating and upregulating *CNOT2*. *CNOT2* decreases the expression of p53, p21, ER/RXR and increases MID1IP1, c-Myc, VEGF, Snail, Slug, Twist, has-miR-3613-5p, has-miR-3916, S27L, RPL37, L31, RPL4, RPL40. RPL5 RPL11, and RPS14 reduce *CNOT2* expression. c-Myc and MID1IP1 elevate *CNOT2* expression.

Downregulated Genes by *CNOT2*	Upregulated Genes by *CNOT2*	Genes Downregulating *CNOT2*	Genes Upregulating *CNOT2*
p53	MID1IP1	RPL5	c-Myc
p21	c-Myc	RPL11	MID1IP1
ER/RXR	VEGF	RPS14	
	Snail		
	Slug		
	Twist		
	has-miR-3613-5p		
	has-miR-3916		
	S27L		
	RPL37		
	L31		
	RPL4		
	RPL40		
